# Human dental pulp stromal cell conditioned medium alters endothelial cell behavior

**DOI:** 10.1186/s13287-018-0815-3

**Published:** 2018-03-21

**Authors:** M. A. Gharaei, Y. Xue, K. Mustafa, S. A. Lie, I. Fristad

**Affiliations:** Department of Clinical Dentistry, Faculty of Medicine, University of Bergen, Årstadveien 19, N-5009 Bergen, Norway

## Abstract

**Background:**

Angiogenesis is of utmost importance for tissue regeneration and repair. Human dental pulp stromal cells (hDPSCs) possess angiogenic potential, as they secrete paracrine factors that may alter the host microenvironment. However, more insight into how hDPSCs guide endothelial cells (ECs) in a paracrine fashion is yet to be obtained. Therefore, the current study aimed to investigate the effect(s) of conditioned medium derived from hDPSCs (hDPSC-CM) on EC behavior *in vitro*.

**Methods:**

hDPSCs were harvested from third molars scheduled for surgical removal under informed consent. The angiogenic profile of hDPSC-CM was identified using human angiogenesis antibody array and enzyme-linked immunosorbent assay (ELISA). Using real-time reverse transcription-polymerase chain reaction (RT-PCR) and ELISA, the mRNA and protein expression level of specific angiogenic biomarkers was determined in human umbilical vein endothelial cells (HUVECs) exposed to hDPSC-CM. The effect of hDPSC-CM on HUVEC attachment, proliferation and migration was evaluated by crystal violet staining, MTT, transwell migration along with real-time cell monitoring assays (xCELLigence; ACEA Biosciences, Inc.). A Matrigel assay was included to examine the influence of hDPSC-CM on HUVEC network formation. Endothelial growth medium (EGM-2) and EGM-2 supplemented with hDPSC-CM served as experimental groups, whereas endothelial basal medium (EBM-2) was set as negative control.

**Results:**

A wide range of proangiogenic and antiangiogenic factors, including vascular endothelial growth factor, tissue inhibitor of metalloproteinase protein 1, plasminogen activator inhibitor (serpin E1), urokinase plasminogen activator and stromal cell-derived factor 1, was abundantly detected in hDPSC-CM by protein profiling array and ELISA. hDPSC-CM significantly accelerated the adhesion phases, from sedimentation to attachment and spreading, the proliferation rate and migration of HUVECs as shown in both endpoint assays and real-time cell analysis recordings. Furthermore, Matrigel assay demonstrated that hDPSC-CM stimulated tubulogenesis, affecting angiogenic parameters such as the number of nodes, meshes and total tube length.

**Conclusions:**

The sustained proangiogenic and promaturation effects of hDPSC-CM shown in this *in vitro* study strongly suggest that the trophic factors released by hDPSCs are able to trigger pronounced angiogenic responses, even beyond EGM-2 considered as an optimal culture condition for ECs.

## Background

The dental pulp, whose main function is to maintain tooth homeostasis, is often susceptible to trauma and bacterial insults [1]. The integrity of the pulp tissue is threatened during pulpal damage and inflammation, which in turn can negatively affect healing and tissue repair in this low-compliant microenvironment lacking collateral blood supply [2].

Regenerative endodontics, an emerging domain of tissue engineering, aims at renewing damaged/lost pulp tissue, implementing principles of cell biology and engineering rather than conventional endodontic treatments [3]. The discovery of postnatal dental pulp progenitor cells, exhibiting similar properties to bone marrow mesenchymal stem cells (MSCs) [4], has encouraged attempts to regenerate the damaged pulp tissue and/or maintain its functional integrity [2, 3]. These precursor cells have high regenerative potential when activated in the damaged pulp and exert paracrine/trophic effects that can alter the host microenvironment [5].

Successful pulp tissue regeneration relies on rapid development of a local microvascular network, providing sufficient oxygen and nutrients to the cells, particularly after tissue injury [6, 7]. The blood vessel network in dental pulp, as elsewhere in the body, is dependent on highly orchestrated angiogenic signaling mechanisms under normal as well as pathological conditions [8]. Angiogenesis, defined as sprouting of new capillaries from preexisting blood vessels [9], plays a pivotal role in wound healing and tissue repair [10]. Angiogenesis is a complex dynamic process, regulated by a sequence of molecular and cellular interactions involving basement membrane and extracellular matrix (ECM) degradation, endothelial cell proliferation, migration, tube formation and maturation into functional blood vessels. A balanced interplay of proangiogenic and antiangiogenic signaling cues, such as matrix metalloproteinases, growth factors, enzymes, cytokines, chemokines, and adhesion molecules, is required during blood vessel formation and development [9].

Human dental pulp stromal cells (hDPSCs) are highly angiogenic and have the potential to induce tissue vascularization via at least two distinct mechanisms; either by secreting angiogenic factors (paracrine effect) that enhance vascularization by local endothelial cells, or by differentiating into vascular endothelium via a process mimicking developmental vasculogenesis [11]. Recently, a growing number of studies have reported that DPSCs release proangiogenic and antiangiogenic proteins under different culture conditions, affecting different steps in the angiogenic process [12]. hDPSCs are also capable of promoting tube formation in human umbilical vein endothelial cells (HUVECs) both in a paracrine fashion and in a coculture system *in vitro* [5]. In agreement with these studies, we have shown that local administration of secretome from hDPSCs, grown under hypoxic conditions, promoted bone healing during distraction osteogenesis and reduced healing time through blood vessel formation [13].

Despite the previously demonstrated proangiogenic effects of human dental pulp cells (hDPCs) both *in vitro* and *in vivo*, the results are to some extent contradictory. Detailed cellular mechanisms by which hDPSCs exert their paracrine angiogenic effects and their potential therapeutic applications in dental tissue engineering are yet to be elucidated. As the angiogenesis is dependent on coordinated signaling by several soluble factors and the effects of growth factors that exert little or no action alone can be greatly augmented by other growth factors [14, 15], studies concerned with the effects of single soluble factors are insufficient to fully address angiogenesis. Therefore, the present study evaluated the angiogenic properties of hDPSC conditioned medium (hDPSC-CM), as a cocktail of soluble factors, by identifying its angiogenic protein profile and ability to affect functional aspects of angiogenesis *in vitro*.

## Methods

### Cell isolation and maintenance

hDPSCs were isolated as described previously [4]. Briefly, human adult third molars, scheduled for routine extraction, were collected from healthy patients aged 18–25 years under informed consent, in accordance with the protocol approved by the Ethical Research Committee at the University of Bergen, Norway (2009/610REK vest). hDPSCs were isolated from pulp tissue by enzymatic dissociation. Single-cell suspension was cultured and maintained in Dulbecco’s modified Eagle’s medium (DMEM; PAA, GE Healthcare, Little Chalfont, UK) supplemented with 10% fetal bovine serum (FBS), 4 mM l-glutamine, 100 U/ml penicillin and 100 μg/ml streptomycin. Cells at passages 3–8 were used for all experiments. hDPSCs from different donors were phenotypically characterized using flow cytometry as described previously by Al-Sharabi et al. [16].

Human umbilical vein endothelial cells (HUVECs) were purchased from Lonza (Clonetics, Walkersville, MD, USA) and expanded in Endothelial Growth Medium-2 (EGM-2) (Lonza). Cells below passage 5 were used for further experiments.

### Collection of conditioned medium from hDPSCs

hDPSCs were seeded at a density of 5000–6000 cells/cm^2^ in culture flasks and grown until approximately 70–80% confluent. Cells were washed three to five times with PBS and incubated with freshly added serum-free DMEM containing penicillin–streptomycin for 48 h at 37 °C in 5% CO_2_. Supernatant was then collected, centrifuged at 4 °C at 3000 × *g* for 3 min followed by 5 min at 1500 × *g*, filtered through 0.2-μm filters and stored in aliquots at −80 °C as hDPSC-CM [13].

### Angiogenic protein profile of CM

hDPSC-CM derived from at least three donors was screened for the relative expression of 55 different angiogenic proteins by Human Angiogenesis Antibody Array kit (Proteome profiler™; R&D Systems, Minneapolis, MN, USA). Briefly, hDPSC-CM was mixed with a detection antibody cocktail and then incubated with a nitrocellulose membrane spotted with capture antibodies in duplicate. Protein detection antibodies were visualized using streptavidin–HRP and chemiluminescent detection reagents. The Gel Doc EZ System (Bio-Rad Laboratories, CA, USA) was used for imaging. Positive signals (mean dot pixel density) on developed membranes were quantified using Quality One image analysis software version 4.7 (Bio-Rad Laboratories).

The expression of selected angiogenic factors in collected hDPSC-CM was quantified using ELISA. Vascular endothelial growth factor A (VEGF-A), stromal cell-derived factor 1 (SDF-1) and insulin-like growth factor 1 (IGF-1) ELISA kits were used according to the manufacturer’s protocol (R&D Systems). Absorbance was measured with a microplate reader (BMG Labtech, Ortenberg, Germany).

### Cell morphology and attachment

Endothelial growth medium (EGM-2) was, based on pilot experiments, set as the optimal culture condition for HUVECs. A dilution of 25% and 50% *v*/v hDPSC-CM in EGM-2 (referred to as 25% CM and 50% CM) was used to evaluate its angiogenic properties. Endothelial basal medium (EBM-2) containing 2% FBS and GA-1000 (Gentamicin, Amphotericin-B) served as negative control in all experiments.

HUVECs were seeded in 96-well plates and allowed to attach for 15, 30, 45 and 60 min at 37 °C in 5% CO_2_. Attached cells were fixed with 4% paraformaldehyde (PFA) and stained with 0.1% crystal violet. Cell morphology was examined with an inverted optical microscope (Nikon Eclipse Ti, Tokyo, Japan). The cell number and cell surface area (freehand drawing) were quantified using ImageJ software (NIH, Bethesda, MD, USA) [17].

### Adhesion kinetics and proliferation

After cell titration, the optimum cell density was chosen as 3000 cells/well. Thereafter, the effect of hDPSC-CM on viability, adhesion and proliferation of endothelial cells was assessed in real-time by the xCELLigence system E-plate 16 (ACEA Biosciences, Inc.) for 72 h at 15 min intervals and the cell status was expressed as the cell index (CI), as described previously [18]. Cell adhesion kinetics and proliferative responses were determined by calculating the mean CI value and slope of the line between two defined time points.

A 3-(4,5-dimethylthiazol-2-yl)-2,5-diphenyltetrazolium bromide (MTT; Sigma Chemicals) assay was performed to validate the previous results in an endpoint experiment. Briefly, HUVECs were seeded at a density of approximately 9000 cells/cm^2^ in flat-bottom 96-well plates in four different culture media. At 24, 48 and 72 h, cells were washed with PBS and incubated with 100 μl MTT working solution (Sigma Chemicals) for 4 h at 37 °C. Then an equal volume of DMSO containing 6.25% (*v*/v) 0.1 M NaOH was added to dissolve the formed formazan for 20 min at room temperature. The optical density was measured at a wavelength of 570 nm with a FLUOstar Omega reader (BMG Labtech).

### Real-time reverse transcriptase polymerase chain reaction

Total RNA was extracted from cultures at 2, 3 and 7 days using a Cell RNA isolation kit (Maxwell^®^; Promega, Madison, WI, USA) and reverse transcribed using a high-capacity cDNA reverse transcription kit (Applied Biosystems, Carlsbad, CA, USA). TaqMan gene expression assays (Applied Biosystems) were used to detect mRNA levels of glyceraldehyde-3-phosphate dehydrogenase (GAPDH), antigen Ki67 (Ki67), proliferating cell nuclear antigen (PCNA), VEGF-A, angiopoietin 1 (Ang-1), angiopoietin 2 (Ang-2), platelet endothelial cell adhesion molecule 1 (PECAM-1/CD31), von Willebrand factor (vWF), platelet-derived growth factor (PDGF) and fibroblast growth factor (FGF) (Table 1). Data were analyzed by a comparative CT method with GAPDH as endogenous control.

**Table 1:** Real-time polymerase chain reaction primers

*Gene*	*Assay ID*	*Amplicon length (base pairs)*
*GAPDH*	Hs99999905-m1	124
*MKi67*	Hs01032443-m1	66
*PCNA*	Hs99999177-g1	69
*VEGF-a*	Hs00900055-m1	59
*ANGPT1*	Hs00375822-m1	74
*ANGPT2*	Hs01048042-m1	95
*PECAM-1*	Hs00169777-m1	65
*vWF*	Hs00169795-m1	79
*PDGFB*	Hs00966522-m1	56
*FGF2*	Hs00266645-m1	82

### Enzyme-linked immunosorbent assay

To further evaluate the real-time reverse transcriptase polymerase chain reaction (RT-PCR) results at the protein level, cell supernatants from each experimental group were collected at 2, 3 and 7 days. The expression of human VEGF and Ang-1 in supernatants was then quantified using ELISA kits according to the manufacturer’s protocol (R&D Systems). Absorbance was measured at 450 nm with a FLUOstar microplate reader (BMG Labtech).

### Migration and microvascular network formation

Cell migration was monitored in real-time by the xCELLigence system CIM-plate 16 (ACEA Biosciences, Inc.) [18]. Each well of the lower chamber was filled with 25% CM or 50% CM as experimental groups, whereas EBM-2 containing 2% FBS and EGM-2 containing 10% FBS served as negative and positive controls, respectively. A total number of 30,000 HUVECs was added to each well of the upper chamber (PET microporous membrane, 96-well format) in EBM-2 containing 2% FBS. Using real-time cell analysis (RTCA) software, a run of 24 h with automatic readings every 15 min was programmed. CI curves were analyzed by calculating the mean value and slope between specific time points.

Endothelial cell migration was also evaluated in a Transwell system. The wells of flat-bottom 24-well plates were filled with experimental and control groups, as already described. Eventually, HUVECs were seeded at a density of 150,000 cells/cm^2^ on cell culture inserts (8-μm pore size, PET track-etched membrane, 24-well format; Falcon, BD, USA) in EBM-2 containing 2% FBS. Following 24 h of incubation, transmigrated HUVECs were fixed with 4% PFA and stained with DAPI. Five representative (fields) images per insert were taken with an inverted phase-contrast microscope (Nikon Eclipse Ti, Tokyo, Japan). The number of positively stained DAPI nuclei was counted manually and by ImageJ software (NIH) [17].

The effect of hDPSC-CM on tubulogenesis was examined in a tube formation assay [13]. HUVECs were cultured in four different culture conditions, at a density of 4.5 × 10^5^/cm^2^ in flat-bottom 96-well plates coated with growth factor-reduced Matrigel basement membrane matrix (BD Biosciences, San Jose, CA, USA). Two representative pictures (40× magnification) per well were taken after 6–8 h with an inverted phase-contrast microscope (Nikon TS100, Tokyo, Japan). The number of nodes, meshes, segments and total tube length were quantified using ImageJ analyzer.

### Statistical analysis

Continuous data are presented as the mean with standard error of the mean (SEM). Multiple comparisons were tested by one-way analysis of variance (ANOVA) followed by a post-hoc Tukey test using the SPSS Statistics version 21.0 (IBM). For real-time analyses of proliferation and migration, a generalized linear model, assuming normal distributed data for comparisons of mean levels and slope, was set up. These analyses were performed using Stata version 14 (StataCorp), and robust variance estimates, adjusting for the repeated/clustered nature of the data, were applied. Statistical significance was determined at *p < 0.05*. All experiments were performed three times in four replicates and verified by at least three hDPSC donors.

## Results

### Proangiogenic and antiangiogenic proteins secreted by hDPSCs

**Fig. 1:**
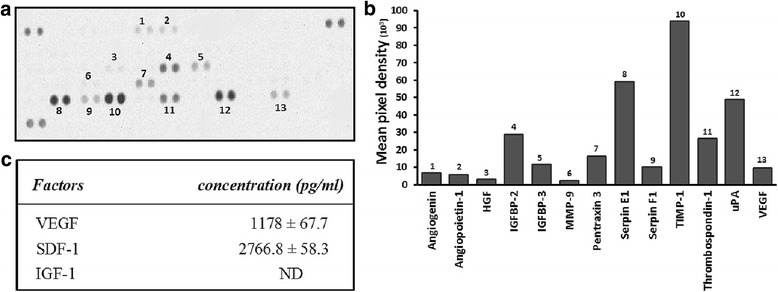
Angiogenic protein profile of human dental pulp stromal cell conditioned medium (hDPSC-CM). **a** Proangiogenic and antiangiogenic proteins detected by human angiogenesis antibody array, marked 1–13 on the array membrane. **b** Histogram generated by quantification of mean spot pixel density using image analysis software. Number on each column corresponds to the protein spotted on the membrane in (**a**). **c** ELISA results showing concentration of vascular endothelial growth factor (VEGF), stromal cell-derived factor 1 (SDF-1) and insulin-like growth factor 1 (IGF-1) in hDPSC-CM**.** Data presented as mean ± standard deviation. ND not detected, HGF hepatocyte growth factor, IGFBP-2,3 insulin-like growth factor binding protein 2 and 3, MMP-9 matrix metalloproteinase 9, TIMP-1 tissue inhibitor of metalloproteinase 1, uPA urokinase plasminogen activator

Thirteen proangiogenic and antiangiogenic proteins were identified in hDPSC-CM (Fig. 1a, b). Tissue inhibitor of metalloproteinase 1 (TIMP-1), plasminogen activator inhibitor (PAI-1)/serpin E1, urokinase plasminogen activator (uPA), insulin-like growth factor binding protein 2 and 3 (IGFBP-2 and 3), pentraxin 3, serpin F-1 and VEGF (among all) were abundantly secreted in hDPSC-CM. No positive detection was found in DMEM, serving as control (data not shown). ELISA revealed high expression of VEGF-A and SDF-1 in hDPSC-CM, whilst IGF-1 was not detected (Fig. 1c). The expression level of detected proteins was highest in hDPSC-CM derived from passage 4 and declined gradually up to passage 8 (data not shown). Consequently, hDPSC-CM from passages 4–6 was used in all experiments.

### hDPSC-CM enhanced HUVEC adhesion and proliferation

**Fig. 2:**
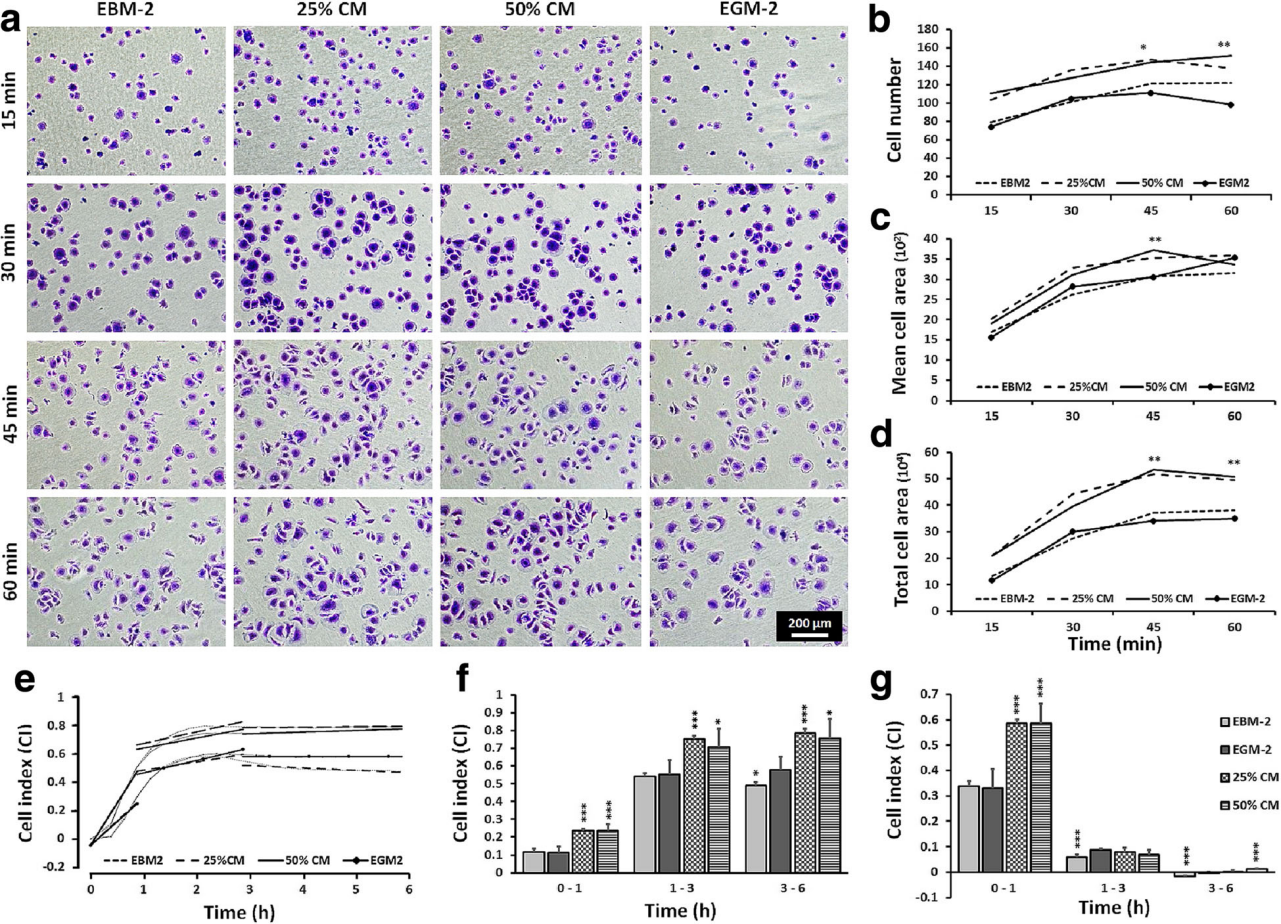
Human umbilical vein cord endothelial cell (HUVEC) morphology, attachment, spreading and adhesion kinetics. **a** Representative crystal violet staining images of HUVECs grown under different culture conditions and time points. **b–d** Graphs from image analysis of attached HUVECs showing cell number (**b**), mean cell area (**c**) and total cell area (**d**). **e** Cell adhesion kinetics monitored in real time using xCELLigence system reveal faster increase in mean cell index (CI) in HUVECs exposed to human dental pulp stromal cell conditioned medium (hDPSC-CM). The effect was independent of concentration and happened within minutes after seeding. **f, g** Representative graphs comparing HUVEC attachment, showing difference in mean CI value (**f**) and slope of the line (**g**) between each selected time period (0–1, 1–3 and 3–6 h). Endothelial growth medium (EGM-2) group served as reference for comparison. Data presented as mean ± SEM. *p ≤ 0.05, **p ≤ 0.01, ***p ≤ 0.001, *n* = 4. EBM-2 Endothelial Basal Medium-2, CM conditioned medium, EGM-2 Endothelial Growth Medium-2

Morphological changes, including initial bond formations and subsequent spreading, were observed to happen faster in response to hDPSC-CM compared with other groups (Fig. 2a). The number of adhered cells as well as the cell spreading area remained higher in the CM groups at all time points, with significant values measured at 45 and 60 min, as shown in crystal violet staining assay (Fig. 2b–d). The CI curves based on real-time xCELLigence readings also confirmed that adhesion steps in HUVECs incubated with CM proceeded more than twice as fast as the controls within the first hour of cell monitoring (Fig. 2e–g).

**Fig. 3:**
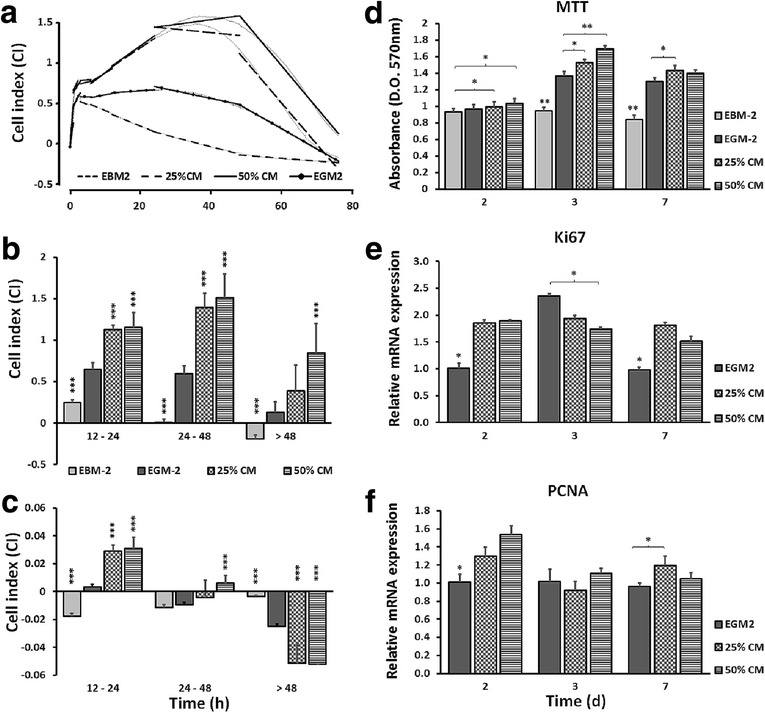
Human umbilical vein cord endothelial cell (HUVEC) proliferation. **a** Cell proliferative activity monitored in real time using xCELLigence system. **b, c** Representative graphs showing difference in mean cell index value (**b**) and slope of the line (**c**) between each selected time period (12–24, 24–48 and 48–72 h). HUVECs treated with human dental pulp stromal cell conditioned medium (hDPSC-CM), independent of concentration, proliferated significantly faster than other groups at all time periods. **d** MTT assay showing significantly enhanced proliferation of HUVECs in hDPSC-CM compared with EGM-2 at 3 days. **e, f** mRNA expression of the proliferative biomarkers antigen KI-67 (Ki67) and proliferating cell nuclear antigen (PCNA) in HUVECs at 2, 3 and 7 days. Endothelial growth medium (EGM-2) group served as reference for comparison. Data presented as mean ± SEM. *p ≤ 0.05, **p ≤ 0.01, ***p ≤ 0.001, *n* = 4. EBM-2 Endothelial Basal Medium-2, CM conditioned medium, EGM-2 Endothelial Growth Medium-2, MTT 3-(4,5-dimethylthiazol-2-yl)-2,5-diphenyltetrazolium bromide, d days

RTCA data, collected over a period of 72 h, showed that HUVECs proliferated significantly faster when grown in the presence of hDPSC-CM compared with controls (Fig. 3a–c). Consistently, CM significantly enhanced the proliferation rate of HUVECs at 72 h as shown by MTT assay (Fig. 3d). However, there was no difference in growth patterns and cell behavior among the CM groups.

At the gene level, the mRNA expression of genes associated with proliferation (Ki67 and PCNA) was significantly upregulated in HUVECs exposed to CM (independent of CM concentration) at 2 and 7 days (Fig. 3e, f).

### hDPSC-CM altered angiogenic gene expression in HUVECs

**Fig. 4:**
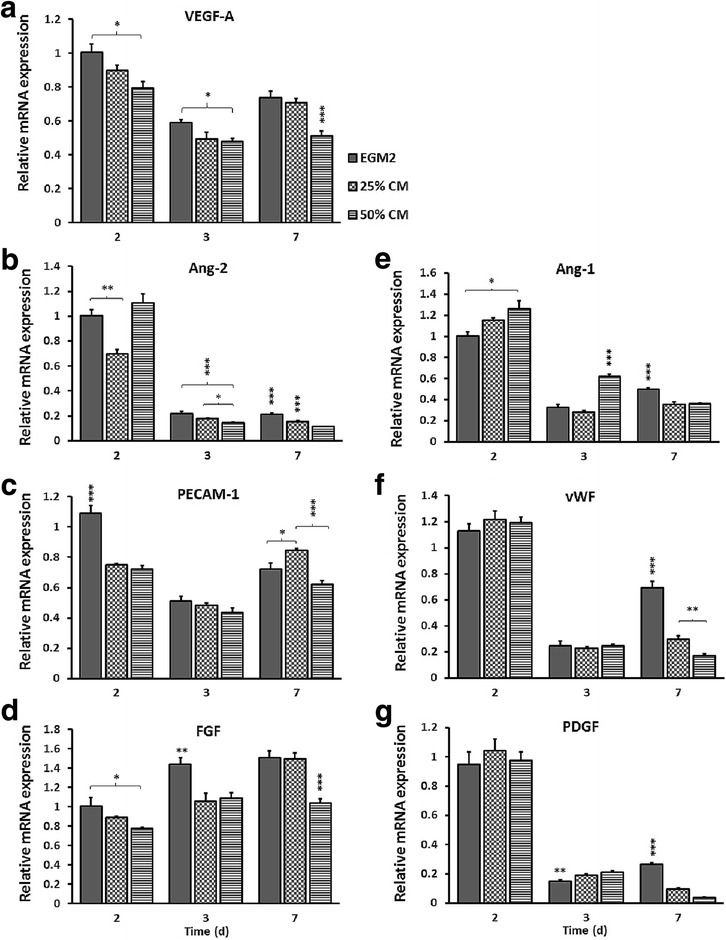
Real-time reverse transcription-polymerase chain reaction (RT-PCR). **a–g** Representative graphs showing expression of the angiogenesis-related genes vascular endothelial growth factor A (VEGF-A) (**a**), angiopoietin 2 (Ang-2) (**b**), platelet endothelial cell adhesion molecule 1 (PECAM-1) (**c**), fibroblast growth factor (FGF) (**d**), angiopoietin 1 (Ang-1) (**e**), von Willebrand factor (vWF) (**f**) and platelet-derived growth factor (PDGF) (**g**) in human umbilical vein cord endothelial cells (HUVECs) grown under different culture conditions at 2, 3 and 7 days. Data normalized to glyceraldehyde-3-phosphate dehydrogenase (GAPDH) as housekeeping gene and presented as mean ± SEM. *p ≤ 0.05, **p ≤ 0.01, ***p ≤ 0.001, *n* = 4. CM conditioned medium, EGM-2 Endothelial Growth Medium-2, d days

The expression of genes encoding vascular development, including VEGF-A, Ang-2, FGF and PECAM-1 was generally decreased at all time points in HUVECs exposed to hDPSC-CM compared with EGM-2 (the higher the concentration of CM, the lower the mRNA expression) (Fig. 4a–d). In contrast, the mRNA expression levels of Ang-1, vWF and PDGF were increased in CM groups compared with EGM-2 at 2 and 3 days, whereas a significant downregulation for the same genes was observed in CM groups at 7 days (Fig. 4e–g).

### hDPSC-CM altered angiogenic protein expression in HUVECs

**Fig. 5:**
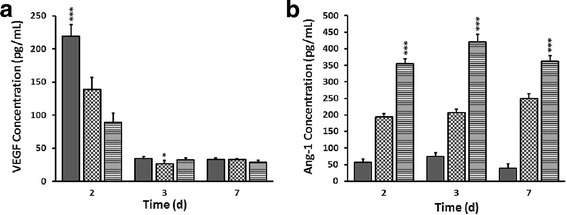
Enzyme linked immunosorbent assay (ELISA). **a, b** Representative graph showing expression of vascular endothelial growth factor A (VEGF) (**a**) and angiopoietin 1 (Ang-1) (**b**). Data presented as mean ± standard deviation. *p ≤ 0.05, ***p ≤ 0.001, *n* = 4. d days

The expression of VEGF was reduced in the groups exposed to CM at 2, 3 and 7 days, following the same pattern as found at gene level (Fig. 5a). Ang-1 expression was consistently and significantly higher in CM groups compared to EGM-2, with 50% CM showing the highest expression level at all time points (Fig. 5b).

### hDPSC-CM stimulated HUVEC migration and microvascular network formation

**Fig. 6:**
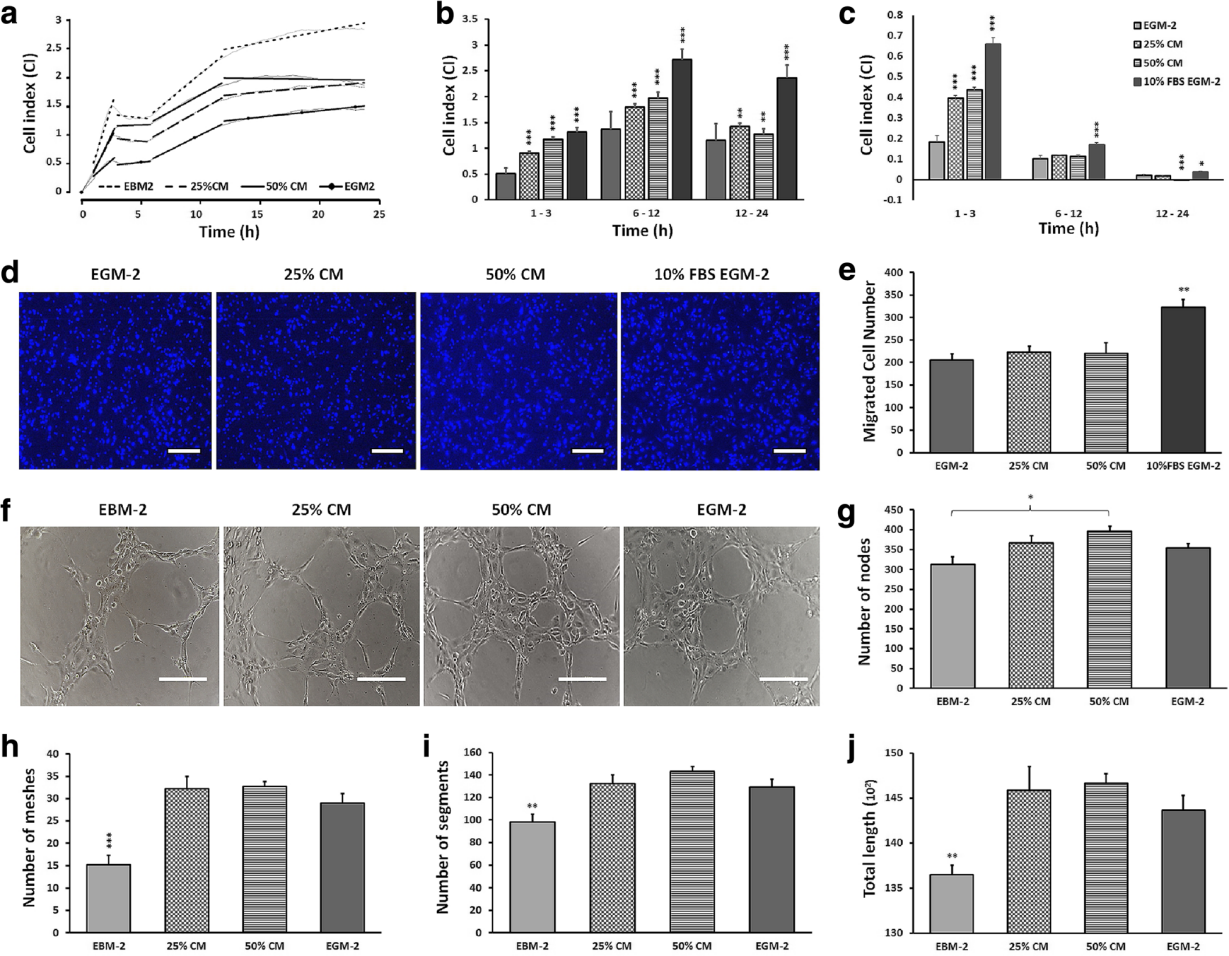
Human umbilical vein cord endothelial cell (HUVEC) migration and microvascular network formation. **a** Representative graph based on real-time measurements showing HUVEC migration rate. **b, c** Graphs showing mean cell index value (**b**) and slope (**c**) between each selected time period (1–3, 6–12, 12–24 h). HUVECs transmigrated significantly faster toward human dental pulp stromal cell conditioned medium (hDPSC-CM) compared with endothelial growth medium (EGM-2), independent of time period and concentration. **d** DAPI staining of transmigrated HUVECs toward different culture conditions at 24 h (10% FBS EGM-2 used as positive control). **e** Graph representing mean migrated cell number. **f** Representative photomicrographs of HUVEC tube formation 6–8 h after incubation in the different culture conditions. **g–j** Graphs demonstrating tube formation parameters, including number of nodes (**g**), number of meshes (**h**), number of segments (**i**) and total tube length (**j**). Data presented as mean ± SEM. *p ≤ 0.05, **p ≤ 0.01, ***p ≤ 0.001, *n* = 4. Scale bar 200 μm. EBM-2 Endothelial Basal Medium-2, CM conditioned medium, EGM-2 Endothelial Growth Medium-2, FBS fetal bovine serum

The CI curves obtained from the xCELLigence platform indicated that HUVECs migrated almost twice as fast toward the hDPSC-CM compared with EGM-2 (Fig. 6a–c). Transwell migration assay also showed that more (although not significant) HUVECs migrated toward CM compared with EGM-2 at 24 h. As expected, positive control containing 10% FBS attracted a significantly higher number of cells through the inserts (Fig. 6d, e).

hDPSC-CM and EGM-2 demonstrated increased endothelial tube formation (Fig. 6f), and network formation parameters were significantly higher compared with EBM-2. Although not significant, a slightly higher number of nodes, segments, meshes and total tube length was quantified in response to hDPSC-CM (Fig. 6g–j).

## Discussion

Targeting therapeutic angiogenesis for pulp tissue regeneration and repair requires stimuli that favor an angiogenic environment. Angiogenesis is tightly regulated in a spatiotemporal manner, with a rigorous interplay between a variety of proangiogenic and antiangiogenic molecules [8, 19]. The role of hDPSC-CM in promotion or inhibition of vessel formation, including its potential effects on EC behavior, is of high importance for optimizing dental pulp regeneration. It is well documented that cultured MSCs secrete trophic and immunomodulatory factors including angiogenic, mitogenic, antiapoptotic, immunomodulatory, chemoattractant and extracellular matrix proteins [20, 21]. However, the secreted molecules and their concentrations differ according to cell type, culture condition and duration [22]. In line with previous reports, the present study clearly demonstrated that a wide range of angiogenic proteins were expressed in hDPSC-CM. Interestingly, regardless of concentration, hDPSC-CM showed the ability to promote EC adhesion, proliferation, migration and tubular network formation, indicating angiogenic stimulation.

Currently used endpoint assays for evaluating angiogenesis do not provide continuous real-time data for cell adhesion, proliferation and migration, and demand high user input [23]. In order to obtain more detailed information we used the highly accurate RCTA platform (xCELLigence RTCA DP, ACEA), facilitating a non-invasive label-free real-time monitoring of ECs dynamics. The interaction of cells with the microelectrodes integrated in the bottom of an E-plate generates an impedance response measured as the cell index (CI). The CI is an arbitrary value proportional to the number of cells, cell morphology and cell adhesion dynamics [18, 24]. In the current study, an exponential increase in the CI readout was observed within minutes when HUVECs were grown in the presence of CM derived from hDPSCs. This remained significantly higher during the first hour after cell seeding, suggesting that CM contains biofactors that enhance HUVEC adhesion kinetics. Cell adhesion and spreading were also visually evaluated and quantified using phase-contrast microscopy after crystal violet staining. Quantitative data showed significantly higher number and surface area for the cells treated with CM compared with cells in EGM-2 alone. Furthermore, changes in cellular morphology within the initial steps of the adhesion process happened earlier than and twice as fast as in the EGM-2 group. As ECM composition can alter endothelial cell spreading dynamics, including the rate and morphology changes [25], it is suggested that the soluble ECM proteins present in CM are responsible for the effects observed. Previous studies have shown that cells on high densities of ECM proteins tend to spread isotropically [26], and that MSC-CM promotes the adhesion ability of HUVECs, possibly through SDF-1 expressed by MSCs [27].

In an attempt to address whether hDPSC-CM could promote HUVEC proliferation and growth, a series of experiments were performed. HUVECs treated with CM showed a significant increase in CI over a period of 72 h, which correlated well with a statistical increase in cell proliferation. The mean CI and the slope of growth curves were calculated for all time periods and the CI for CM-treated cells, irrespective of concentration, remained higher than that for untreated cells. The metabolic activity levels, as measured by MTT, were also significantly higher in the CM groups at 72 h. In parallel, the mRNA levels of the cell proliferation markers Ki67 and PCNA were significantly upregulated in the cells exposed to CM at 48 h, suggesting that the increase in proliferative activity of HUVECs from genome to transcriptome happened in a coordinated temporal manner. The enhanced proliferation rate might be explained by the abundant release of mitogens and antiapoptotic factors, such as VEGF [28], angiopoietins [19], TIMP-1 [29] and uPA [30], and their possible synergistic effect(s) in hDPSC-CM. As shown previously, DPSCs secrete VEGF, a key regulatory factor of vascular permeability and angiogenesis, which enhances the survival and differentiation of ECs [31]. VEGF signaling also promotes EC proliferation, filopodial extensions and ECM degradation [9]. uPA enhances endothelial permeability through intracellular signaling pathways shared with VEGF [30, 32]. TIMP-1 has a well-established antiapoptotic activity in many different cell types including HUVECs and possesses a novel MMP-independent cytokine-like activity in the regulation of cell proliferation [29]. In addition, MSCs derived from bone marrow and adipose tissue have the capacity to increase EC proliferation [33–35]. In contrast, other studies have shown no effect of CM from hDPSCs and BM-MSCs on EC growth [12, 36]. A possible explanation could be insufficient serum concentration for cell survival in these experimental setups.

There is a body of evidence that ECs sense small alterations in the microenvironment and have a remarkable capacity to adjust to the local requirements, which in turn determines the direction of differentiation [37]. Vascular development is initiated by Ang-2 and VEGF promoting proliferation, migration and sprouting of ECs, whereas establishment of functional and stabilized vessels is facilitated by Ang-1 in a competitive Tie2 receptor binding with Ang-2 [38]. The expression of endothelial specific markers was altered in the presence of CM, with a downregulation of VEGF-A, Ang-2 and FGF at all time points. As further confirmed by ELISA, the extracellular VEGF secretion followed the same pattern as the gene expression. The substantial proliferation and survival of HUVECs for up to 3 days in culture might suggest a high level of antiapoptotic and mitogenic proteins present in CM, suppressing excessive gene expression in a feedback loop mechanism. In addition, increased permeability and nascent phenotype of ECs could be attributed to the downregulation of PECAM-1 in CM-treated cells. Proliferation and differentiation of cells are two processes that do not occur simultaneously. In order for nascent vessels to mature into tubes with controlled permeability, the proliferation of ECs should subside [39]. As discussed earlier, CM induced EC proliferation for a prolonged period. Thus, it is logic to speculate that the differentiation of cells was delayed accordingly. An upregulation of Ang-1 expression, observed at day 2, is possibly mediated through competitive receptor binding with Ang-2. Nevertheless, the significant increase in Ang-1 protein expression in HUVECs exposed to CM compared to EGM-2 is indicative of EC maturation or vascular stabilization at 3 and 7 days. The translational process and protein turnover can take some time, independent of the translation/degradation rate and half-life of different proteins, as the cells need to adapt to microenvironmental changes [40, 41]. This delay might explain possible discrepancies between gene and protein expression at 7 days for Ang-1.

A further key step in angiogenesis is the migration of proliferating ECs along a gradient of chemotactic stimuli through the disintegrated basement membrane and ECM in the remodeled perivascular space. Data from the RTCA chemotactically driven migration setup showed that HUVECs migrated significantly faster through microporous inserts toward the media derived from hDPSCs than EGM-2. Higher concentrations of CM (50% CM) provided the strongest chemotactic stimuli for the recruitment of HUVECs. Despite a higher number of migrated HUVECs toward CM than EGM-2 alone, as shown in the 24 h endpoint migration assay, the difference was not found to be significant. This suggests that RTCA provides a more accurate platform as the electrodes sense passing cells in real time by recording CI changes. On the other hand, abundant secretion of stromal derived factor 1 (SDF-1) and VEGF in hDPSC-CM in our experiments might be the key chemotactic stimuli for HUVEC migration [12, 42, 43]. SDF-1 is involved in hematopoietic and nonhematopoietic stem/progenitor cell migration and homing [43], and the SDF-1α–CXCR4 axis plays a crucial role in the recruitment of CXCR4-positive DPSCs toward the damaged sites [44]. VEGF is (partly) responsible for the chemotactic activities of hDPSCs, as shown by neutralizing VEGF in the CM from hDPSCs [12].

Tube-like structure and lumen formation by migrated ECs is critically important in healing and regeneration. The Matrigel tube formation assay indicated a higher vessel density and larger lumen areas in CM groups than those in EGM-2. Although the number of nodes, meshes and tube length were higher in groups exposed to CM, the results were not significant. However, studies have previously reported that CM from different sources of MSCs or their side populations have the ability to promote tube formation [5, 45, 46], mostly through VEGF signaling that plays a pivotal role in EC sprouting and tubular network formation. This was evidenced by a reduction in the tubular perimeters after addition of VEGF and/or FGF neutralizing antibodies [10].

## Conclusion

It is important to note that hDPSCs, also defined as dental pulp fibroblasts, are a heterogeneous cell population that can regulate the pericellular microenvironment by secreting ECM, growth factors and growth factor binding proteins. The angiogenic/vasculogenic properties of hDPSCs are independent of direct contact and are mainly due to the paracrine activity of the secreted soluble factors. This *in vitro* study strongly demonstrated that CM released from hDPSCs is able to trigger pronounced angiogenic effects, even beyond EGM-2 considered to be an optimal culture condition for ECs. Therefore, the valuable effect of hDPSC-CM on angiogenesis either alone or in combination with EGM-2 might be a result of the additive or synergistic action of several growth factors. This synergistic effect of growth factors in CM is likely to be important in therapeutic applications requiring angiogenesis. As a better understanding of the dynamic and mechanisms underlying the effects of CM develops, a clearer role of CM in vascular development and maintenance evolves. However, thorough proteomic analyses to detect detailed concentrations of identified angiogenic proteins in hDPSC-CM and novel *in vivo* studies are warranted to strengthen this conclusion, and to evaluate the potential effect of CM in healing and regeneration.

## Abbreviations

Ang-1: Angiopoietin 1
Ang-2: Angiopoietin 2
CI: Cell index
CM: Conditioned medium
DMEM: Dulbecco’s Modifies Eagle’s Medium
DMSO: Dimethyl sulfoxide
EBM-2: Endothelial Basal Medium-2
EC: Endothelial cell
ECM: Extracellular matrix
EGM-2: Endothelial Growth Medium-2
ELISA: Enzyme-linked immunosorbent assay
FBS: Fetal bovine serum
FGF: Fibroblast growth factor
GAPDH: Glyceraldehyde-3-phosphate dehydrogenase
hDPSC: Human dental pulp stromal cell
HUVEC: Human umbilical vein endothelial cell
IGF-1: Insulin-like growth factor 1
IGFBP-2: 3 Insulin-like growth factor binding protein 2 and 3
Ki67: Antigen KI-67
MMP-9: Matrix metalloproteinase 9
MSC: Mesenchymal stem cell
PAI-1: Plasminogen activator inhibitor
PCNA: Proliferating cell nuclear antigen
PDGF: Platelet-derived growth factor
PECAM-1: Platelet endothelial cell adhesion molecule 1
PFA: Paraformaldehyde
RTCA: Real-time cell analysis
RT-PCR: Real-time reverse transcriptase polymerase chain reaction
SDF-1: Stromal-derived factor 1
TIMP-1: Tissue inhibitor of metalloproteinase 1
uPA: Urokinase plasminogen activator
VEGF: Vascular endothelial growth factor
vWF: Von Willebrand factor
